# Interventricular Septal Hematoma after Retrograde Intervention for a Chronic Total Occlusion of a Right Coronary Artery: Echocardiographic and Magnetic Resonance Imaging—Diagnosis and Follow-Up

**DOI:** 10.1155/2016/8514068

**Published:** 2016-01-14

**Authors:** Makoto Araki, Tadashi Murai, Yoshihisa Kanaji, Junji Matsuda, Eisuke Usui, Takayuki Niida, Sadamitsu Ichijo, Rikuta Hamaya, Tsunekazu Kakuta

**Affiliations:** Department of Cardiology, Tsuchiura Kyodo General Hospital, Ibaraki 300-0053, Japan

## Abstract

The reverse CART technique provides the potential to modify the retrograde procedure by improving the controlled movement of the retrograde wire and improve the success rates of percutaneous coronary intervention (PCI) of chronic total occlusion (CTO). Development of interventricular hematoma is a rare complication of CTO PCI. A 63-year-old man with effort angina with a right coronary artery CTO lesion underwent PCI by retrograde approach from the LAD to a septal branch. A contrast “stain” was demonstrated surrounding the septal collateral channel used for the retrograde approach at the end of the procedure without symptom. Echocardiography indicated an increased interventricular septum thickness with low echo signals region and decreased contractility. Cardiac magnetic resonance (CMR) imaging using gadolinium showed a diffusely thickened septum with a low signal fusiform neocavitation delimited by an enhanced-signal ring suggesting intraventricular septal dissecting hematoma. After conservative treatment, follow-up echocardiogram and CMR showed the resolution of the hematoma without clinical events. This case highlights the potentially lethal complication of septal perforator dissection and hematoma that may cause severe myocardial injury caused by retrograde approach for CTO PCI.

## 1. Introduction

The reverse CART technique has given the potential to modify the retrograde procedure by improving the controlled movement of the retrograde wire without advancing the balloon retrogradely, which potentially reduces the septal channel injury and improves the success rates [1]. In this report, we would like to present a case of interventricular septal hematoma after retrograde intervention for a chronic total occlusion and the chronological change of echocardiographic and magnetic resonance imaging.

## 2. Case Report

**Figure 1:**
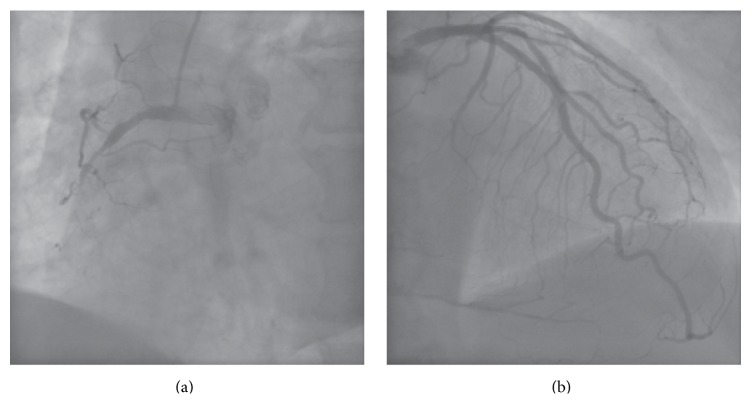
Diagnostic coronary angiography. (a) Chronic total occlusion of right coronary artery (RCA). (b) Retrograde filling of the distal RCA from the left anterior descending artery (LAD).

**Figure 2:**
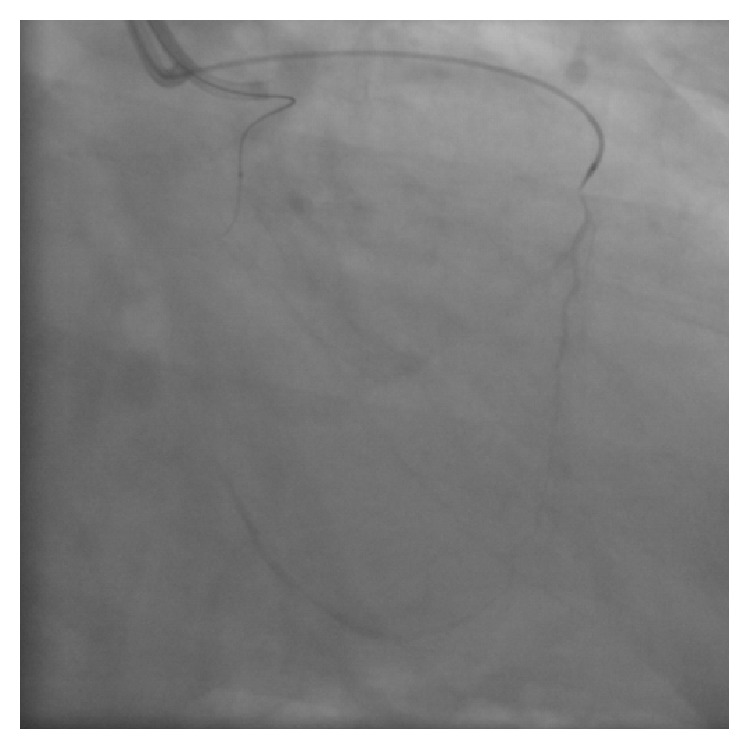
Retrograde filling of the RCA via a septal perforator from the LAD.

**Figure 3:**
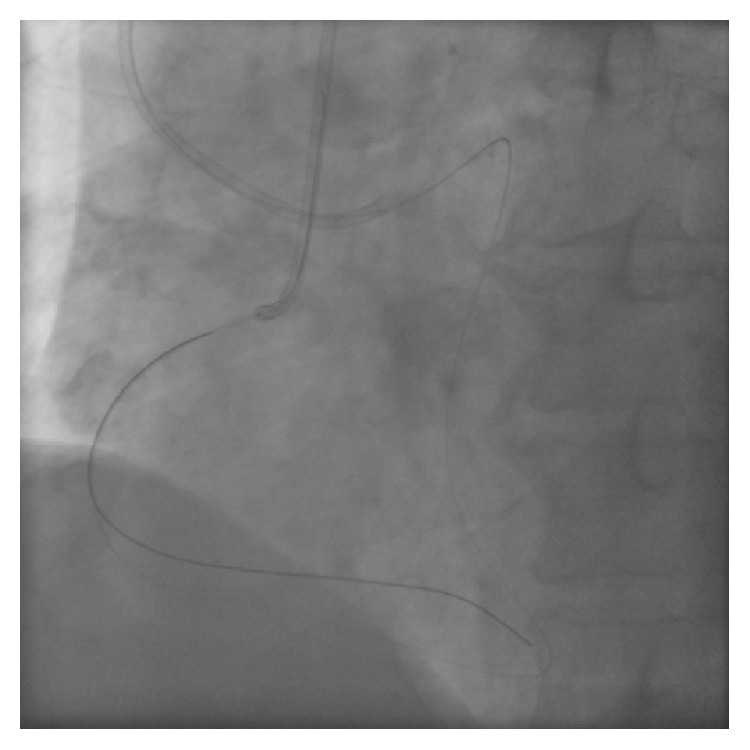
Retrograde guidewire was passed from the subintimal space to reach the proximal RCA true lumen and into the antegrade guiding catheter.

**Figure 4:**
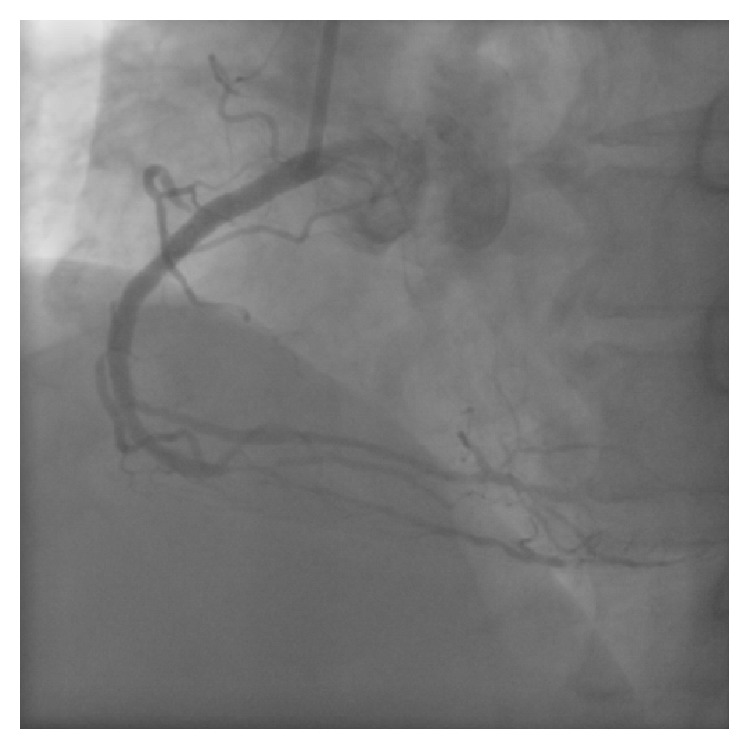
Final angiogram of RCA.

A 63-year-old man with effort angina was found to have a chronic total occlusion (CTO) of the mid right coronary artery (RCA) by coronary CT examination. Diagnostic coronary angiography confirmed the RCA CTO to be 20 mm long (Figure 1(a)). Retrograde filling of the distal RCA occurred via a septal perforator from the left anterior descending artery (LAD) (Figure 1(b)). After discussion, the patient opted for percutaneous coronary intervention. An initial antegrade approach was unsuccessful to negotiate the CTO lesion. A subsequent attempt was made to treat the CTO via a retrograde approach.  Figure 2 demonstrates retrograde filling of the RCA. A floppy hydrophilic guidewire (Fielder FC, Asahi Intecc, Japan) was successfully advanced via the septal perforator from the LAD to the posterior descending branch of the RCA. However, the microcatheter advancement of a Corsair extra-support catheter (Asahi Intecc, Japan) into the distal RCA posterior descending artery was extremely difficult because of tortuosity of the collateral channel. Because of the anticipated difficulty to advance a balloon to RCA via retrograde approach, the retrograde guidewire was advanced into the occluded segment and the recanalization by the reverse controlled antegrade and retrograde tracking (CART) under intravascular ultrasound guidance (IVUS) was attempted. Following the balloon inflation on antegrade guidewire creating the subintimal connection, the retrograde guidewire was easily passed from the subintimal space to reach the proximal RCA true lumen under IVUS guidance and into the antegrade guiding catheter (Figure 3). Subsequently, the retrograde guidewire was trapped by the inflation balloon in the antegrade guiding catheter, and the microcatheter was easily advanced through the distal portion of RCA into the antegrade guiding catheter. The guidewire was exchanged for a 330 cm guidewire (RG3, Asahi Intecc, Japan) through the microcatheter and externalization was completed. The CTO lesion was dilated by using retrograde wire to deliver the balloon antegradely, and three drug-eluting stents were successfully implanted (Figure 4).

**Figure 5:**
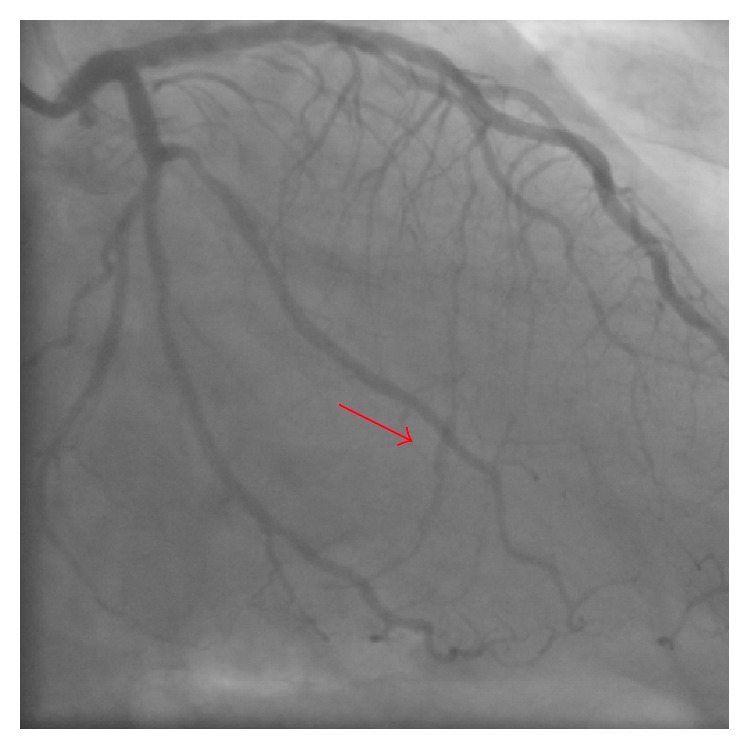
A contrast “stain” surrounding the septal collateral channel used for the retrograde approach at the end of procedure (red arrow).

During the procedure, the patient remained hemodynamically stable and asymptomatic. A contrast “stain” was demonstrated surrounding the septal collateral channel used for the retrograde approach at the end of the procedure (Figure 5).

**Figure 6:**
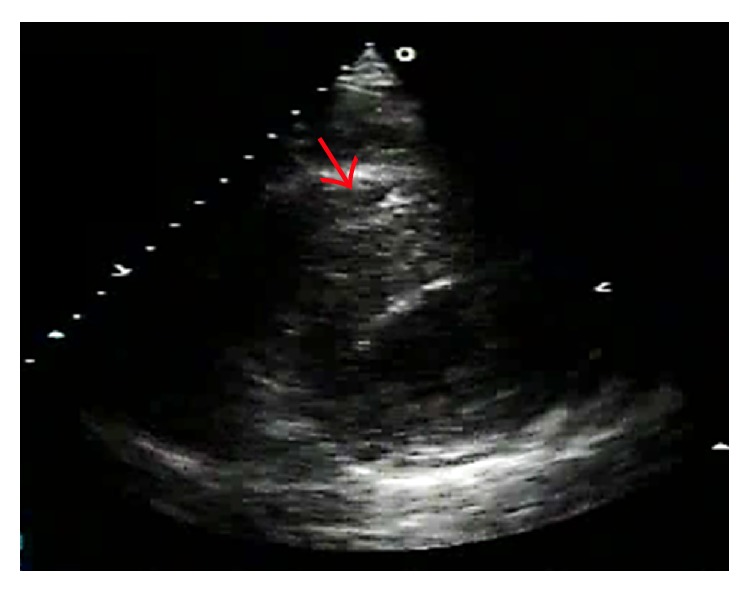
Increased interventricular septum thickness and abnormal motion by postprocedural echocardiography.

**Figure 7:**
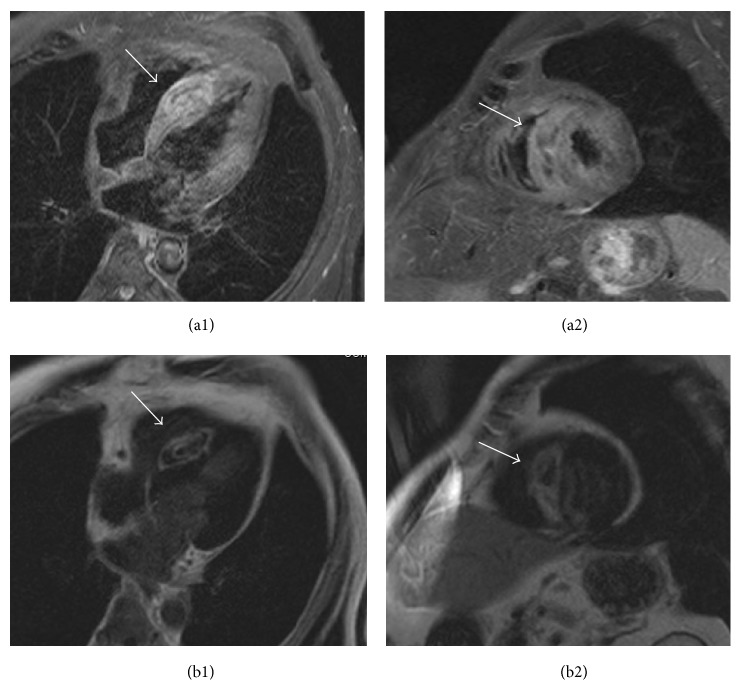
Interventricular septum with hematoma accompanied with microvascular obstruction by cardiac magnetic resonance (CMR) imaging performed 6 days after the index PCI. (a) T2-weighted image. (b) Late gadolinium enhancement (LGE) image.

**Figure 8:**
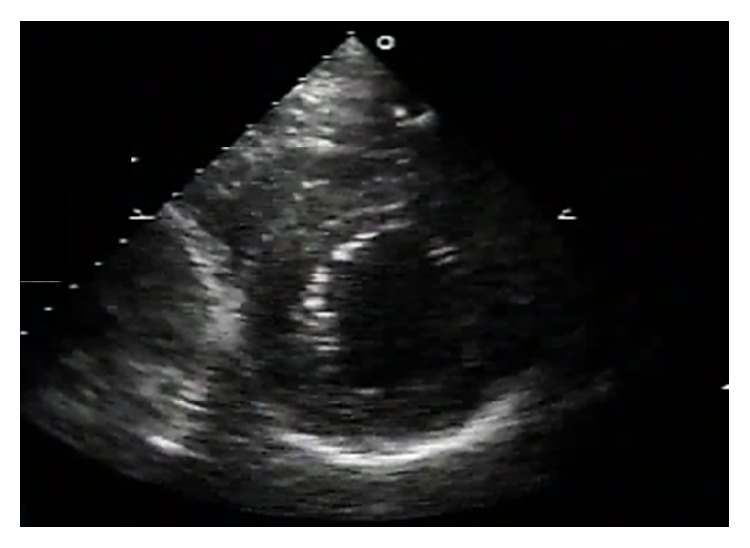
Echocardiography at 6-week follow-up demonstrating the resolution of the hematoma.

**Figure 9:**
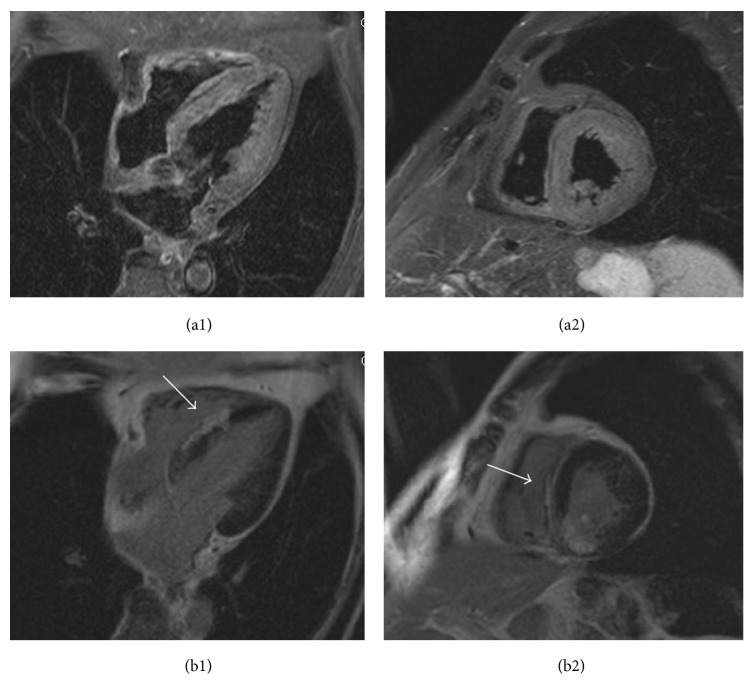
CMR at 4 months later demonstrated resolution of the hematoma with epicardial LGE. (a) T2-weighted image. (b) LGE image.

Postprocedure echocardiography demonstrated an increased interventricular septum thickness with low echo signals core and abnormal motion (Figure 6). Postprocedural peak CK-MB was 151 IU/L. Cardiac magnetic resonance (CMR) imaging performed 2 weeks later showed a diffuse widening and hypokinetic interventricular septum with a low signal fusiform neocavitation (hematoma) delimited by an enhanced-signal ring after administration of gadolinium due to myocardial fibrosis (Figures 7(a) and 7(b)). These findings confirmed the diagnosis of intraventricular septal dissecting hematoma. After conservative treatment, the patient was discharged uneventfully. Echocardiogram at 6 weeks later demonstrated spontaneous hematoma resolution (Figure 8). CMR at 4 months later showed the resolution of the hematoma with epicardial late gadolinium enhancement (Figures 9(a) and 9(b)).

## 3. Discussion

Development of interventricular hematoma is a rare complication of percutaneous coronary intervention for chronic total occlusion (CTO) [2]. Previously, septal hematoma formation has been described after childhood VSD repair [3, 4] and cardiac resynchronization therapy defibrillator implantation [5]. Spontaneous resolution of a ventricular septal hematoma and VSD after retrograde CTO coronary intervention was also reported [6]. In the present case, artery dissection caused by guidewire manipulation in septal perforators probably led to intraventricular myocardial hematoma. Although the patient had no chest pain and had uneventful in-hospital clinical course, ventricular arrhythmia was observed and potential lethal adverse cardiac event might have emerged. In a previous report, CMR images indicating an accidental contrast agent deposition into the interventricular septum complicating left ventriculogram and subsequent myocardial infarction in a patient with suspected coronary artery disease have been reported [7]. The reported CMR and echocardiographic findings were consistent with the findings of the present case, indicating spontaneously resolved hematoma and residual late gadolinium enhancement. There was no report describing a large hematoma complicating with CTO PCI, and this case highlights the potentially lethal complication of septal perforator dissection and hematoma that may cause myocardial injury. Caution should be taken when crossing septal perforators using a soft guidewire with extra-support microcatheters because these have the potential to create significant injury to the ventricular septum. We also have to recognize a potential myocardial injury by CTO PCI which may present a large septal hematoma after PCI completion without symptom.
